# Chromosome abnormalities and the genetics of congenital corneal opacification

**Published:** 2011-06-17

**Authors:** A. Mataftsi, L. Islam, D. Kelberman, J.C. Sowden, K.K. Nischal

**Affiliations:** 1 Clinical and Academic Department of Ophthalmology (CADO), Great Ormond Street Hospital, London, U.K.; 2 IInd Department of Ophthalmology, Aristotle University of Thessaloniki, Thessaloniki, Greece; 3 Developmental Biology Unit, Institute of Child Health, University College London, London, U.K.; 4 Ulverscroft Vision Research Group, UCL Institute of Child Health, London, U.K.

## Abstract

Congenital corneal opacification (CCO) encompasses a broad spectrum of disorders that have different etiologies, including genetic and environmental. Terminology used in clinical phenotyping is commonly not specific enough to describe separate entities, for example both the terms Peters anomaly and sclerocornea have been ascribed to a clinical picture of total CCO, without investigating the presence or absence of iridocorneal adhesions. This is not only confusing but also unhelpful in determining valid genotype-phenotype correlations, and thereby revealing clues for pathogenesis. We undertook a systematic review of the literature focusing on CCO as part of anterior segment developmental anomalies (ASDA), and analyzed its association specifically with chromosomal abnormalities. Genes previously identified as being associated with CCO are also summarized. All reports were critically appraised to classify phenotypes according to described features, rather than the given diagnosis. Some interesting associations were found, and are discussed.

## Introduction

Congenital corneal opacities (CCO) occur with a prevalence of 6/100,000 newborns in Europe [1]. The causes are complex, and include genetic and pre- and peri-natal environmental factors. To date controversy exists on the exact genetic causes of CCO with some of this controversy muddied by inaccurate clinical phenotyping [2]. This issue of phenotyping is crucial if we are to progress in our understanding of the heterogeneous genetic causes of this severe condition, which frequently causes blindness from birth, and its association with other anterior segment developmental anomalies (ASDA) of the eye.

In simplistic terms, the embryology of the ocular anterior segment structures can be summarized as the formation of the bi-layered optic cup from the forebrain neuroectoderm, the invagination and separation of the lens vesicle from the overlying surface ectoderm, and the anterior migration of surrounding mesenchymal progenitor cells mainly of neural crest origin to participate in the formation of the anterior segment tissues, the cornea, the iris, and the iridocorneal angle. When the tissue destined to become the cornea fails to separate from the underlying tissues, or fails to form properly with the appropriate differentiated cell content and under the influence of specific growth factors generated by the surrounding tissues, then the resulting cornea lacks the structure and function to maintain transparency and appears as a CCO [3]. Elucidating the genetic factors that normally orchestrate this process is crucial for the understanding why CCO can occur due to DNA mutation and/or environmental factors.

According to the Online Mendelian Inheritance In Man Database (OMIM) the term sclerocornea is commonly used to describe a congenital malformation of the cornea, such that the boundary between the cornea and the sclera is obscured (OMIM 269400). Usually the involvement is limited to the peripheral part of the cornea but it may extend to the entire cornea, so-called sclerocornea totalis. Peters anomaly according to OMIM consists of a central corneal leukoma (an opaque white spot), absence of the posterior corneal stroma and Descemet membrane, and a variable degree of iris and lenticular attachments to the central aspect of the posterior cornea [4] (OMIM 604229). Various degrees of iridocorneal and/or keratolenticular adhesion including Peters anomaly and peripheral scleralization of the cornea were first described as “anterior chamber cleavage syndrome” by Reese and Ellsworth in 1966 [5]. More recently terms like anterior segment dysgenesis (ASD), or ASDA have been used instead to refer to a broad spectrum of disorders affecting the anterior segment, including Axenfeld Rieger syndrome, congenital glaucoma, congenital cataract, anterior segment mesenchymal dysgenesis, and corneal plana and aniridia, all of which may exhibit corneal pathology [6].

Genes thus far implicated in CCO include paired box 6 (*PAX6*), pituitary homeobox 2 (*PITX2*), forkhead box C1 (*FOXC1*), forkhead box E3 (*FOXE3*), beta 1,3-galactosyltransferase-like (*B3GALTL*), and keratocan (*KERA*), indicating the genetic heterogeneity of the condition (Table 1). Dominant mutations of *PAX6, PITX2, FOXC1, FOXE3*, and recessive mutations of *B3GALT*L and *KERA* have been identified as the cause of ASDA including CCO in some patients. Although none have been identified that cause a significant proportion of cases of CCO. Chromosomal abnormalities have also been implicated in CCO, including some that do not incorporate these genes. Identifying a chromosomal anomaly that is associated with a particular phenotype provides a rapid way of localizing at least one area on the genome that may harbor a gene(s) contributing to that condition. This has proved to be a very useful method in revealing the genetic causes of numerous monogenic diseases.

**Table 1:** Genes known to be implicated in CCO (in humans).

**Locus**	**Gene**	**ASDA or other ocular feature associated with this gene**	**Key references**	**OMIM**
1p32	FOXE3* (forkhead box E3)	ASDA+cataract, primary aphakia	[6], [7]	*601094, 610256
2p22-p21	*CYP1B1*	congenital glaucoma, Peters anomaly	[8], [9], [10]	*601771, 604229
3q26.3–27	*SOX2*	anophthalmia, microphthalmia, coloboma, optic nerve hypoplasia	[11], [12]	*184429
4q25	PITX2* (paired-like homeodomain transcription factor 2; formerly RIEG1)	Axenfeld-Rieger anomaly/syndrome, Peters anomaly	[13]	*601542
6p24	*TFAP2A*	colobomatous microphthalmia, corneal clouding, buphthalmos	[14], [15]	*107580
6p25	*FOXC1* (forkhead box C1; formerly *FKHL7*)	glaucoma, ad iridogoniodysgenesis	[16]	*601090
8q13.3	EYA1* (eyes absent 1)	CCO, ASDA, congenital cataract	[17]	*601653
10q25	PITX3*	ad ASDA+cataract	[18]	+602669
10q26	FGFR2* (fibroblast growth factor receptor 2)	Peters anomaly, Axenfeld-Rieger anomaly	[19], [20]	*176943
11p13	PAX6* (paired box gene 6)	congenital cataracts, anophthalmia, aniridia, ad Peters anomaly, CNS defects (in one family), microcornea and microphthalmia	[21], [22], [23], [24]	*607108
12q21.3	DCN* (décorin)	ad Congenital Stromal Corneal Dystrophy (CSCD)	[25]	610048, 125255
12q22-q23	KERA* (keratocan)	CNA2, ar cornea plana with sclerocornea	[26], [27]	*603288, 217300
13q12.3	B3GALTL* (beta-1,3-glucosyltransferase)	ar Peters-plus (Kivlin) syndrome	[28], [29]	261540, *610308
16q22–23	MAF* (transcription factor)	ad cataract and PA and microphthalmia (1 family), cataract and microcornea and iris coloboma (1 family)	[30], [31]	*177075
18q21.3	*RAX* (homeobox gene)	R anophthalmia and L S-CCO with persistent fetal vasculature and retinal detachment	[32]	*601881
20p11.2	VSX1*	posterior polymorphous corneal dystrophy, keratoconus	[33]	122000, *605020
20p13-p12	SLC4A11*	ar congenital hereditary endothelial dystrophy (CHED2)	[34]	217700

The asterisk indicates genes without known associated chromosome abnormality associated with CCO but for which point mutations or small insertions or deletions have been identified. “ad” and “ar” indicate autosomal dominant and autosomal recessive inheritance.

We undertook a comprehensive review of the literature to evaluate the type of CCO and the associated genetic causes, particularly the range of chromosomal anomalies reported thus far. We classified the phenotypic entities by the described features and not necessarily by the terminology used by the authors, as classification and nomenclature varies and this is confusing when trying to establish phenotype-genotype correlations.

**Figure 1:**
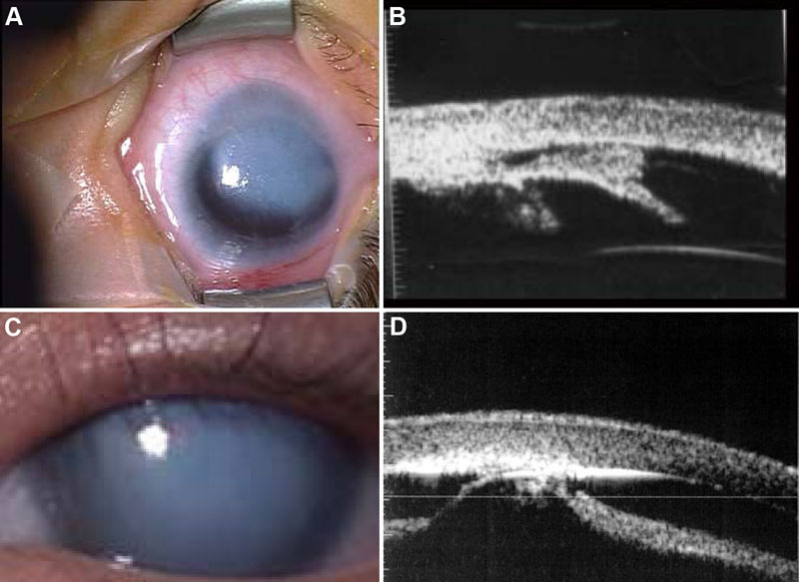
Iridocorneal and keratolenticular adhesions. Peters anomaly (PA) classically describes a congenital corneal opacity resulting from an iridocorneal (**A**, **B**) or keratolenticular adhesion (**C**, **D**). The opacity may be central (**C**, **D**) and may take up a small part or the whole of the cornea, or may be eccentric (**A**, **B**). It is unreliable to ascribe this term judging only from the clinical picture without making use of ultrasonography, notably ultrasound biomicroscopy (UBM; **B**, **D**), which helps visualize the structures of the anterior segment and prove or disprove the presence of adhesions (**B**, **D**). The use of UBM in **A** and **B** shows contact between the cornea and iris (iridocorneal adhesion) and in **C** and **D** contact between the cornea and the anterior lenticular surface (keratolenticular adhesions), resulting in disorganization and loss of clarity in the central cornea, confirming the diagnosis of PA. NB. These opacities are avascular (see Figure 3) and in these cases the lens is of a normal size.

**Figure 2:**
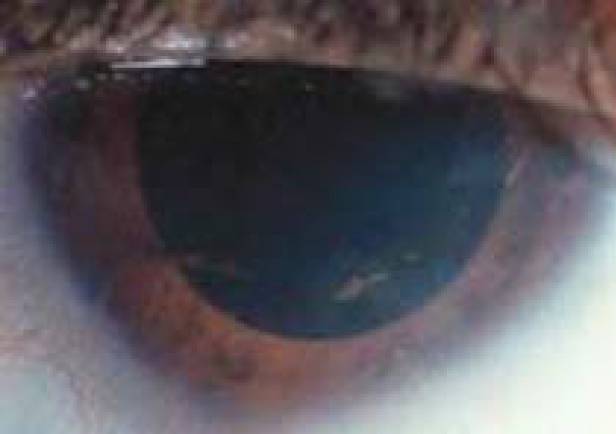
Sclerocornea. This is a case of sclerocornea, i.e., peripheral scleralization of the cornea, accompanied by the feature of cornea plana (CNA2, OMIM 217300) which in this review we designate S-CNA to distinguish it from total corneal opacification (S-CCO, see Figure 3**A**,**B**). In S-CNA the anterior segment is otherwise normal.

**Figure 3:**
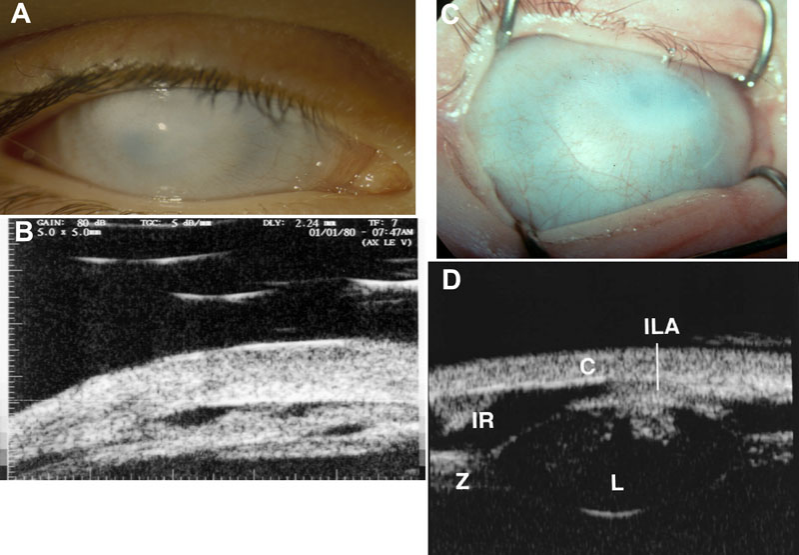
Total corneal opacification. Two typical examples of the phenotype designated as total congenital corneal opacification. S-CCO (**A**-**D**). This condition confusingly, has been previously described clinically as sclerocornea in the literature. However, anterior segment imaging using UBM shows that the lens has failed to form normally in case **A** and **B** and that in case **C** and **D** there is failure of the lens (L) to separate from the cornea (ILA) and there is an abnormal zonular ciliary complex (Z); this suggests another primary lens problem leading to a secondary CCO. Note both opacities are vascularized.

All primary developmental CCOs were included in our search, i.e., those apparently resulting from a default that occurs during the complex developmental process of anterior segment formation. In the literature the term sclerocornea is often confusingly used to describe total corneal opacification, where the status of anterior segment structures is not known. Wherever possible we assigned the CCOs to the subcategories of Peters anomaly (PA), sclerocornea with cornea plana (S-CNA), and total “sclerocornea” (total opacification of the cornea: S-CCO) based on the clinical features described in each paper. The designation PA was only used when there was evidence or description of iridocorneal or keratolenticular adhesion resulting in central or eccentric, localized or total corneal opacification (Figure 1). S-CNA was only designated as such if the phenotype was typical of Cornea Plana 2, CNA2 (OMIM 217300 [35]; Figure 2). This entity is characterized by peripheral corneal scleralization but a view of the eye through the center of the cornea is possible, and there is always accompanying cornea plana. CNA2 can be caused by recessive mutation in *KERA* encoding the keratan sulfate proteoglycan, keratocan (OMIM 603228), which is important for corneal transparency. S-CCO was designated in cases in which all of the cornea is opaque and resembles the sclera (Figure 3).

Although not classically grouped under ASDA, our search did not specifically exclude those corneal dystrophies that are present from birth, that is congenital hereditary endothelial dystrophy (CHED), posterior polymorphous corneal dystrophy (PPCD), congenital stromal corneal dystrophy (CSCD) and X-linked endothelial corneal dystrophy (XLECD), as they are primary, congenital, and concern the formation of the cornea [36]. We excluded storage disorders such as mucopolysaccharidosis type IV, which can present from birth in the form of CCO, along with all other metabolic causes and storage disorders, as etiologically distinct. Corneal opacification of non-genetic causation, metabolic/storage disorders, and corneal dystrophies with the exception of CHED, PPCD, CSCD and XLECD, were also excluded.

We performed searches on PubMed, using the following strategy:

#1; congenital corneal opacity OR Peters anomaly OR corneal opacity OR corneal opacification OR leukoma 4468
#2; (“chromosome disorders”[MeSH Terms] OR (“chromosome”[All Fields] AND “disorders”[All Fields]) OR “chromosome disorders”[All Fields] OR (“chromosome”[All Fields] AND “abnormalities”[All Fields]) OR “chromosome abnormalities”[All Fields] OR “chromosome aberrations”[MeSH Terms] OR (“chromosome”[All Fields] AND “aberrations”[All Fields]) OR “chromosome aberrations”[All Fields] OR (“chromosome”[All Fields] AND “abnormalities”[All Fields] 35276
#3; anomaly OR abnormality OR deletion OR translocation OR ring OR trisomy OR monosomy 501777
#4; #2 OR #3 AND #1 409

We excluded the articles regarding animal studies or exclusively laboratory work. All remaining articles were reviewed, as well as all relevant references, which were not cited in Pubmed. We applied no language or time period restriction. Search results were cross-referenced with the OMIM, DECIPHER, and London Dysmorphology Databases, by using the term “corneal opacity.” Retrieved articles and their relevant bibliographies were reviewed. Any cases of microphthalmia with severe disruption of the globe and no specific mention of CCO, or cases of CCO attributed to isolated congenital glaucoma, were noted but not included in the detailed analysis of CCO and chromosomal disorders. Reports of mutations were reviewed, but again not included in the analysis hereafter. Finally, for the purposes of this study, it was not possible to include any papers reporting a genotype associated with CCO but without describing the phenotype, or papers discussing a CCO phenotype without a reported genetic abnormality.

## Discussion

In total 254 published studies were collected, of which 101 had no case of human CCO with an associated chromosomal abnormality to include in our analysis, and were therefore excluded. No case of congenital corneal dystrophy came up in the search. The majority of analyzed papers were case reports, small series of patients or familial incidence of CCO, but also a few review articles that summarized reported cases and added new ones, or that were essentially used for cross-checking and retrieving all relevant references. The findings were grouped according to the associated chromosome anomaly for ease of reference. Known monogenic causes of CCO are listed in Table 1, where the references cited are those specifically describing a CCO in association with each gene and other associated phenotypes are also listed.

No studies were found to associate CCO with genetic disruption in chromosomes 7, 15, 16, 19, and 20. BenEzra et al. [37] reported microphthalmia with CCO in a patient and PA in his sibling, both of whom presented unusual sister chromatid disjunction. Wertelecki et al. [38] described a case of bilateral PA associated with abnormal centromere-chromatid apposition (ACCA). Although interesting, this association is not informative for a candidate locus/gene.

### Chromosome X

Xp22.3 syndrome is reported in a series of studies with a similar range of phenotypes. Temple et al. [39] and Al-Gazali et al. [40] first described a clinical syndrome in 1990 comprising linear skin defects at birth, congenital microphthalmia with CCO (MLS – Microphthalmia with Linear Skin defects or MIDAS – Microphthalmia, Dermal Aplasia and Sclerocornea), associated with Xp22.2 chromosome breakpoint or deletion. Ocular phenotypic expression is variable, including severe microphthalmia in the form of orbital cyst, posterior stromal corneal opacity with peripheral synechiae [40], S-CCO [39-41], microcornea [42,43], PA [44], and intrafamilial variable expression [45]. Several papers reporting Xp22 microdeletion with MLS [43,45-50] suggest that severe microphthalmia is often associated with severe disruption of the anterior segment and secondary CCO.

Ropers et al. [51] described a case with a balanced Xp22/3q12 translocation presenting with Aicardi syndrome and right microphthalmic eye with corneal opacity, whereas Donnenfeld et al. [46], in 1990, reported a case with an unbalanced Xp22/3p22 translocation showing features suggestive of Aicardi or Goltz syndrome, together with unilateral microphthalmia and S-CCO. Naritomi et al. [43] presented two cases with Xp22.3 deletion, microphthalmia with corneal opacity, and combined partial phenotypes of both Aicardi and Goltz syndromes. Aicardi syndrome is a triad of callosal agenesis, infantile spasms, and chorioretinal lacunae; Goltz syndrome is focal dermal hypoplasia. Both conditions are described as having X-linked dominant inheritance with in utero lethality in males. In some reported cases only the feature of corpus callosum agenesis is associated with Xp22 deletion and MLS [41,52,49]. It is plausible to assume that these conditions may be associated with a contiguous gene deletion [43], or that clinical overlap can be seen as a result of genetic heterogeneity and variable penetrance [53], or due to any of the following factors: mosaicism, skewed patterns of X-inactivation, and variable viability of mutated cells [42].

Glaucoma in cases of MLS or MIDAS associated with disruption of Xp22.1–3 region has been described [54-56]. All three reports describe sclerocornea in some of the affected cases but Cape et al. describes a variable phenotype ranging from anophthalmia and S-CNA, to total corneal opacity with PA, whereas Mucke et al. clearly show an ectatic right cornea and left S-CCO. In addition, Paulger et al. [57] describe a case of MLS with bilateral microphthalmia and CCO, congenital glaucoma, and lens absence in one eye and thin lens in the other. This suggests that the primary problem is not corneal: in other words, corneal opacification is secondary to a primary intraocular event. This intraocular event may affect the iridotrabecular region (hence the glaucoma cases) or the iris alone (explaining the iridocorneal adhesions and/or the partial aniridia) [55]. It seems reasonable therefore to suggest that this region on the X chromosome may contain genes that affect the growth/development of the iris, its root and/or the trabecular meshwork.

Bleyl et al. [52] report a mother and son with a pericentric inversion of the X chromosome, disturbing the Xp22.3 as well as the Xq27 regions, and presenting with PA and sclerocornea without microphthalmia. In the mother, PA is confirmed histopathologically by the absence of Descemet’s membrane in the cornea, and the presence of iridocorneal adhesions [58]. However the son is diagnosed with complete bilateral S-CCO, as no evidence of iridocorneal adhesions is found on ultrasonography. This latter is not ultrasound biomicroscopy as stated, however the authors do attempt to classify phenotypes according to specific features. In fact, the ultrasound shows no evidence of a normal lens. The phenotype shown by Bleyl et al. [52] (Figure 2B in their article) is that described by our group previously to be caused by a failure of the lens to form properly. It is suggested by the authors that X-linked sex-determining region Y box 3 (*SOX3*), localized close to the Xq27 breakpoint, may possibly be associated with the ocular anomalies. *SOX3* is a gene that promotes neuronal differentiation and is implicated in the development of the midline forebrain structures. No other report as yet has associated it with ocular development, including whole gene deletions and intragenic mutations within the gene [59,60].

Sakurai et al. [61] report a case of mosaic trisomy X associated with unilateral S-CNA in an infant with Dandy-Walker syndrome, together with ipsilateral microphthalmia, persistent pupillary membrane, iris hypoplasia, and bilateral optic disc coloboma. On the other hand, Lloyd et al. [62] present four patients with mosaic Turner syndrome, where ocular findings included trabecular meshwork dysgenesis with congenital glaucoma, and Rieger anomaly. The authors hypothesize that mosaicism causing defective neural crest cell migration may be at the origin of the ocular developmental anomalies.

### Chromosome 1

Steffensen et al. [63] reported a boy presenting with partial trisomy 1q31-qter and small eyes with corneal opacities in association with systemic malformations. No further clinical details are given. There are subsequent reports of partial trisomy 1q32-qter associated with goniodysgenesis and cataract [64] or glaucoma [65], without mention of corneal problems.

David et al. [66] presented a case of bilateral PA and secondary glaucoma with a balanced translocation between chromosomes 1 and 7. The authors suggested that the disrupted genes in the reciprocal areas, transforming growth factor-beta 2 (*TGFbeta2*) in 1q41 and/or histone deacetylase 9 (*HDAC9*) in 7p21, may be contributors to the pathogenesis of PA perhaps through decreased gene dosage. This has not been further investigated in the literature so far.

We found no cases of CCO associated with aberrations in 1p32, which is the locus harboring *FOXE3*. This gene is a forkhead transcription factor that has been implicated in early lens and anterior segment formation, mutations of which have been found in dominantly inherited ASDA and cataract, as well as in recessive primary congenital aphakia with secondary anterior segment dysgenesis [6,7,67]. This locus may not show enough instability for translocations/deletions to happen, or may harbour genes essential for viability of a fetus.

Finally, a single report presents the association of Goldenhar syndrome, including unilateral corneal dermoid, with an unbalanced translocation resulting in 1p36.33 trisomy and 16p13.3 monosomy [68]. The significance of this is unclear.

### Chromosome 2

MacDonald et al. [69] reported a case of partial trisomy 2q32-qter and monosomy of 6p (resulting from a 2:6 translocation) with S-CCO, progressive corneal staphyloma and aniridia. The significance of this case is unclear.

Gambrelle et al. [70] described a case with interstitial deletion of 2q24-q32 and microcephaly, limb malformation, and ocular anomalies. Although microphthalmia is stated to be present, the axial length given is not abnormal for 34 weeks gestation. Microcornea is therefore a possible alternative diagnosis in this case. The corneal opacities mentioned are not described. Similarly, Franceschini et al. present a case with interstitial deletion 2q31q33 as having microphthalmia, but the patient's photograph shows normal palpebral fissure aperture, suggesting that the globes were not small [71]. Again, this could possibly be mislabelled microcornea. The same deletion caused left corneal opacity, mild microphthalmia, and narrow palpebral fissures in the case described by Young et al. the same year [72], and bilateral blepharophimosis, asymmetric microphthalmia in another case presented by Benson et al. [73], but no clinical details are given.

Finally, several other reports, including Boles et al. [74], describe 2q24-q31 deletion presenting with bilateral retinal coloboma, right iris coloboma, mild ptosis, and short palpebral fissures, but without microphthalmia, and the cornea is not commented on. It can be thus concluded that 2q31 harbours a gene important for ocular morphogenesis, but contribution to corneal morphogenesis in particular is not possible to infer from the available data.

Unilateral microphthalmia with PA, as well as multiple systemic malformations, was found in a girl with interstitial deletion 2q14-q21 [75]. Severe developmental anomalies and bilateral microphthalmia with cataract were described in a case with del2q21q24 [76]. Finally, two siblings with bilateral PA, persistent fetal vasculature, and systemic abnormalities were described by Kivlin et al. [77] in 1986, although 2q21 disruption, due to a balanced translocation, was only present in one of the two, and the authors question the association. All these anomalies appear to concern the development of the eye as a whole, rather than the cornea primarily.

Heathcote et al. [78] reported a case of dup2p21-p25 with multiple dysmorphic features presenting with both primary and secondary corneal anomalies: microcornea and thickened Bowman's membrane with glycosaminoglycan deposition, as well as persistent vascular pupillary membrane with corneal focal Descemet's membrane and stroma disruption in areas of attachment.

Interestingly, sine oculis homeobox homolog 3 (*SIX3*), a transcription factor gene expressed in the anterior forebrain and eyes during early vertebrate development, is located in 2p21. It is found mutated in holoprocencephaly, but it also appears that Six3 plays a role in regulating the development of the vertebrate eye anterior segment [79,80].

Furthermore, a locus associated with primary congenital glaucoma lies in 2p21 [81]. Finally, the *CYP1B1* gene, which has been implicated in congenital glaucoma as well as PA, is located in 2p22 (Table 1).

### Chromosome 3

In papers published regarding ASDA and chromosome 3 anomalies, the common area of interest appears to be 3q21–28. Translocations, duplications and trisomies affecting this area lead to variable but severe eye abnormalities ranging from an intracranial rudimentary eye and anophthalmos to microphthalmia with CCO [82-85]. This again suggests that the CCO described here is secondary to globe disruption. The inference is that this region may contain genes that are involved upstream in organization of the developing eye.

One such gene is *SOX2*, located in 3q26.3-q27, mutations in which have been shown to cause anophthalmia and microphthalmia in association with other variable forebrain abnormalities [11,12,86]. *SOX2* codes for a transcription factor that controls the expression of several genes involved in embryonic development, thus being critical for early embryogenesis and for embryonic stem cell pluripotency.

### Chromosome 4

A large number of papers present the association of 4q25 deletion/disruption with features of Axenfeld-Rieger syndrome, the associated corneal pathology usually being prominent Schwalbe's line [87-94]. Some of the cases present associated microphthalmia [87], and/or microcornea [88,92,95-97].

Studies quantifying gene dosage in such patients suggest that the phenotype is the result of *PITX2* haploinsufficiency [94,98,99] or changes in its regulatory elements [100]. *PITX2* (pituitary homeobox 2), formerly *RIEG1*, is a gene on chromosome 4q25 coding for a paired-bicoid homeodomain transcription factor expressed during eye development [101,102]. PITX2 physically interacts with FOXC1, and these two proteins appear to participate in a common pathway, which can explain how altered gene dosage of *FOXC1* can produce a phenotype similar to that found in mutations/deletions of *PITX2* [103].

It is interesting that there have been two reports of PA with a *PITX2* mutation [13,104] but no chromosomal abnormality involving 4q25 has ever reported PA/sclerocornea. The patient described by Arikawa et al. [104] presented unilateral PA, proven by UBM, as part of a whole eye disruption, including a persistent hyaloid artery. It is unclear if the patient described by Doward et al. [2] truly presented PA, as no ultrasonography was performed and no fundal view was possible through the total corneal opacification with peripheral vascularisation. The contralateral eye of the patient showed typical ARA. PA might then be regarded as the very severe end of spectrum of ARA, whereby iridocorneal adhesions are so disruptive for the cornea that it completely opacifies. Vascularisation of the cornea usually indicates some sort of intraocular insult in cases of ASDA.

On the other hand, there has been a case of unilateral PA in association with multiple midline defects and a familial chromosome 4 inversion, affecting 4q12 and 4q13.3, and not 4q25 [105]. This is a unilateral “whole eye” disruption: microphthalmia with anterior dysgenesis, cataract and dysplastic retina.

Finally, two cases combined Rieger's anomaly [106] or PA and microphthalmia [107] with 4p- deletion syndrome (Wolf-Hirschhorn syndrome). Genetic analysis was only done by DNA autoradiography at the time, so there is insufficient information regarding chromosome breakpoints to locate a possible disease-associated gene.

### Chromosome 5

There has only been a single report of iridocorneal adhesions resulting in very mild corneal opacity in a case of partial trisomy 5q [108]. The authors mention defects in Descemet's membrane but they do not explain how this was ascertained, since there is no histopathology report. The significance of this genotype-phenotype correlation is unclear.

### Chromosome 6

*FOXC1* has been shown to play a role in the regulation of embryonic and ocular development. Mutations in this gene cause various glaucoma phenotypes including primary congenital glaucoma, autosomal dominant iridogoniodysgenesis anomaly, and Axenfeld-Rieger anomaly.

There is abundant evidence that deletions affecting the 6p24–25 region bearing *FOXC1* result not only in Axenfeld-Rieger syndrome but also in corneal opacities, the majority of which by far are due to iridocorneal rather than keratolenticular adhesions [109-118]. More severe disruption of the iridotrabecular area may lead to further corneal insults resulting in corneal vascularisation [113]. Again the corneal opacity appears to be a secondary phenomenon. Conversely, a few reports describe 6p deletions in patients without ocular dysgenesis [119,120], suggesting that disruption of *FOXC1* leads to incomplete penetrance of ocular abnormalities.

There is description of bilateral S-CNA in an infant with an interstitial deletion of 6p22.3-p24, presenting with systemic and dysmorphic features but no other ocular pathology [121]. It may be that this primary corneal dysgenesis was due to disruption of *FOXC1*, its regulatory elements, a different gene, or of a combination of these. For example, transcription factor AP-2 alpha (*TFAP2A*) lies in 6p24 and has been found to be mutated in patients with branchio-oculo-facial syndrome who present with colobomatous microphthalmia and corneal clouding among other features, suggesting that *TFAP2A* plays a role in ocular embryogenesis [14,15]. This interesting association remains to be confirmed.

Tabbara et al. [122] described a case of ARA combined with bilateral microcornea, incomplete aniridia, and cataract, in association with a presumptive isochromosome 6. This is an extreme phenotype of ocular dysgenesis, where corneal involvement is part of a “whole eye” disruption.

In the only paper reporting duplication of 6p25, the cornea shows increased central corneal thickness and reduced corneal diameter, while maintaining normal endothelial cell morphology and density [123].

### Chromosome 8

Trisomy 8 mosaicism has been associated with CCO, often unilateral. Photographs of these, where available [124-129], overwhelmingly display a clinical phenotype typical of a corneal dermoid. Only one case [129] did not show this and showed isolated iridocorneal adhesions. Where histopathology was available [126,128], this confirmed features of choristoma. We suggest that an isolated ocular phenotype of corneal dermoid should raise the possibility of a trisomy 8 mosaicism, as systemic features may be very mild. This corneal dermoid is usually very flat with reticular vascularisation.

### Chromosome 9

Of the two reports of ASDA with abnormalities affecting chromosome 9, one is an interstitial deletion between 9q22-q32 [130], and the other is a partial trisomy 9 mosaicism [131]. In this latter report, the individual is affected unilaterally only. Its significance, both because of the mosaicism and the unilaterality, is unclear.

In the report of a deletion 9q22-q32, bilateral S-CNA is reported [130]. Interestingly transforming growth factor beta receptor 1 (*TGFBR1*) is located at 9q22. This is a cell receptor regulating TGFB-induced transcription of target genes, in particular collagen and connective tissue growth factor [132]. One may speculate that disruption of this gene may lead to perturbation of the extracellular matrix homeostasis within the cornea.

On the other hand, there is a recent report by Hanna et al. [133] presenting a patient with unilateral PA, atypical systemic associations, and a heterozygous deletion involving the receptor tyrosine kinase-like orphan receptor 2 (*ROR2*) gene on chromosome 9q22.31, another possibly implicated gene in the case reported by Ying et al. [130].

### Chromosome 10

Partial duplication of 10q has been shown to be associated with congenital glaucoma and S-CCO [134]. In this case, at the time of PKP, total absence of iris was found, leading us to suggest that this entity is total corneal opacification secondary to an intraocular disruption.

Bilateral S-CNA was reported in a boy with an unbalanced translocation involving 17p and 10q chromosomes [135]. Although detailed location of the breakpoints was not possible at the time, it is reasonable to assume that this was another case of CCO resulting from 10q disruption.

It is worth noting that fibroblast growth factor receptor 2 (*FGFR2*) is on chromosome 10q26. Mutations in this gene are known to result in the craniosynostoses Crouzon, Apert, and Pfeiffer syndromes [136]. There has been a report of ARA-like changes in a patient with a *FGFR2* mutation [20], as well as features of PA in another patient [19]. Whether the above duplication results in a very severe ARA-like phenotype resulting in absence of iris is unclear.

Yunis et al. [137] reported partial trisomy for 10q(ter), resulting in bilateral microphthalmia, and bilateral lens opacities with obliteration of anterior chamber. The retina was replaced with fibrous tissue. Again it is worth noting that known mutations in *FGFR2* result in a gain of function.

Also, the transcription factor *PITX3* lies in 10q25 [18,138], and mutations in this gene are associated with “anterior segment mesenchymal dysgenesis” including dense corneal opacification (central or peripheral) with iris adhesion, i.e., Peters anomaly, and cataract.

Finally, discrete peripheral S-CNA with prominent Schwalbe's line, iris strand and iris hypoplasia consistent with the Rieger anomaly phenotype is described in a case of deletion of 10p13 [139].

### Chromosome 11

*PAX6*, on 11p13, is a homeobox gene that is important in regulating the development of the eye. Reports of del11p13 mosaicism, duplication of 11p12–13, del(11)(p13p13), and deletion of 11p13–15, have all shown features typical of those seen in aniridia: hypoplastic iris stroma or absent iris, corectopia, as well as microphthalmos with or without cyst, iridocorneal adhesions with secondary corneal opacity, polar cataract and foveal hypoplasia [140-144].

Of some interest however is a report of interstitial deletion of 11q (either 11q14 or 11q22) resulting in iridocorneal adhesion with secondary CCO in one eye, and complete iridocorneal adhesion in the other eye with secondary CCO and corneal vascularisation [145]. This suggests that on 11q a gene affecting iridocorneal development may also reside.

### Chromosome 12

In a single report chromosome 12p deletion is described in a child with severe S-CCO with advanced glaucoma [146]. That CCO is described with corneal vascularisation. One eye had no evidence of a lens on USS and the other had a lens. The CCO is secondary to intraocular anomalies. No further history was available. This case demonstrated severe glaucoma, anterior segment disruption and severe CCO. It is important to note that as far back as 1970 Zinn had shown in the chick embryo that removal of the developing lens resulted in an opaque cornea [147]. Therefore, the CCO described here is likely to be secondary to lens formation failure.

### Chromosome 13

Association of interstitial deletion of 13q14-q22 with features of Rieger syndrome is suggested based on two reported cases [148]. This is reinforced by a study of a family with Rieger syndrome of variable severity mapping the disease to 13q14 [149]. However, there were no patients with primary corneal pathology in the above studies.

In addition, a sole case with trisomy 13 was reported with PA as part of whole eye disruption and multiple systemic abnormalities [150]. No conclusions can be drawn from this association.

### Chromosome 14

A single report presents the association of 14q22.1–22.3 interstitial deletion and bilateral CCO [151]. The phenotype is not sufficiently described to ascertain if the cornea is primarily or secondarily involved. The authors suggest that bone morphogenetic protein 4 (*BMP4*), previously shown to be mutated in mice with anterior segment abnormalities, may contribute to the phenotype. This association is interesting but needs to be confirmed by further studies. This is probably the case, as, in the same year, Bakrania et al. [152] reported the association of a *BMP4* mutation and anophthalmia-microphthalmia in two distinct families.

### Chromosome 17

A case of 17q12 duplication was reported with sex reversal, PA, microphthalmia, glaucoma, and systemic abnormalities [153]. The pathogenicity of this genetic defect and the relevance of transcription factor 2 (*TCF2*), a gene involved in diabetes-associated phenotypes that lies on the duplicated area, is uncertain.

### Chromosome 18

Trisomy 18 (Edwards' syndrome) has been associated with congenitally opaque corneas with absence of Bowman's and Descemet's membrane, and superficial conjunctival vascularisation, with or without microphthalmia [154,155]. However, the occurrence of normal corneas in other cases has led Mullaney et al. [156] to hypothesize that CCO in the former paper was possibly trophic (secondary) in origin. At the other end of the severity spectrum, a case of primary congenital aphakia and anterior segment aplasia was described by Johnson et al. [157]. It is unsafe to draw conclusions about the nature of CCO in trisomy 18, based on these observations alone.

There has been an association of deletion of 18q with CCO consistent with PA, and multiple systemic anomalies [158], while deletion of 18q21 was reported in a child with bilateral microphthalmia, microcornea, and unilateral inferior corneal opacification [159]. A patient with right anophthalmia and left S-CCO and persistent fetal vasculature, was found to be a compound heterozygote for retina and anterior neural fold homeobox (*RAX*) gene mutations [32]. The *RAX* homeobox gene is on chromosome 18q21.3 and is crucial for the proper formation of the optic vesicle. CCO in the above cases appears to be secondary to whole globe disruption.

Additionally, a recent report associates the development of Fuchs' dystrophy of the cornea with genetic variation in the transcription factor 4 *TCF4* gene, which lies on 18q21.2-q21.32 [160]. This gene encodes the protein E2–2, which is a transcription factor expressed in the developing corneal endothelium and involved in cellular growth and differentiation.

### Chromosome 21

Ring chromosome 21 was reported in a boy with unilateral PA [161]. The unilaterality and mildness of the phenotype suggests that this genetic abnormality had a minor contribution to corneal morphogenesis.

Partial monosomy 21 was reported in a case with bilateral S-CCO [162]. In fact, the karyotype is complex (45,XX,-6,-der6t(6;21)(p25;q22.1)-21 and the translocation present in this patient resulted in a derivative (6;21) chromosome, it is thus unclear if the phenotype can be associated with gene disruption at the breakpoints, the partial monosomy 21, or the partial monosomy 6. An argument to suggest that 21q22 is implicated comes from three reports in the literature associating CCO and 21q22 deletion [163-165]. CCO in these cases ranged in severity, from unilateral to bilateral and combined with severe microphthalmia. Once more, corneal involvement appears to be secondary.

### Chromosome 22

A large series of patients with 22q11.2 deletion syndrome describe common ocular findings, but not of the cornea [166,167]. However, Casteels et al. [168] and Erdogan et al. [169] each described a case of unilateral PA, while Binenbaum et al. [170] showed several cases with a spectrum of anterior segment anomalies, including S-CCO, S-CNA, descemetocele, iridocorneal adhesions (PA), and severe anterior segment dysgenesis. This latter series of patients suggests that a gene/genes affecting anterior segment embryogenesis reside at the 22q11.2 locus. It could be hypothesized that disruption of the genes coding for crystallin beta 1, crystallin beta 2, and crystallin beta 3 (*CRYBB1*, *CRYBB2*, and *CRYBB3*), in 22q11.2, interferes with normal lens formation, and secondarily with anterior segment morphogenesis. The occurrence of cataract is, however, scarce in this syndrome [171], and does not necessarily accompany ASDA.

## Comment

Of the 111 papers included in this study for detailed ocular phenotype analysis and genotype-phenotype association, only 53 show a picture of the affected cornea/anterior segment, while 29 show a picture of the face and 29 present no pictures at all. Histology findings are given in 16 of the 19 cases where corneal transplantation took place or post-mortem autopsy was performed.

Out of the 28 papers where the term “sclerocornea” appears, it was used to describe a CNA-like phenotype in only 13 papers (S-CNA), whereas in the remaining 15 it signified total corneal opacification (S-CCO). Similarly, in 4 of the 17 papers where the term “Peters anomaly” was used, it described a complete corneal opacity, without sonographic or histologic evidence of iridocorneal or keratolenticular adhesions. Ocular ultrasonography was used to better describe the phenotype in only 4 papers [52,85,146,162] and ultrasound biomicroscopy (UBM) in only one [104].

Reviewing the literature on chromosome anomalies and associated CCO highlights how confusion may occur when a clinical phenotype is mislabeled or inaccurately presented (Figure 1 and Figure 3). This confusion may prevent useful genotype-phenotype correlations from being identified and the true nature of this spectrum of diseases and associated genes from being revealed. From the data provided, we attempted to summarize associations regarding primary corneal involvement (Table 2). This information may point to possible candidate genes.

**Table 2:** Chromosome loci associated with CCO without whole globe disruption according to CCO classification used in this review.

**Phenotype**	**Associated loci**	**Genes in the region, known to be associated with ASDA in humans or mice**
CCO with iridocorneal or keratolenticular adhesions (PA)	mosaic trisomy 8 [129]	?
	1q41 or 7p21 [66]	TGFbeta2
		*HDAC9*
S-CNA	6p22.3-p24 [121]	*FOXC1*, *TFAP2A*
	9q22-q32 [130,133]	*TGFBR1*, *ROR2*
flat corneal dermoid	mosaic trisomy 8 [124-128]	?
CCO of unspecified type	Xq27 or Xp22.3 [52]	*SOX3*
	2p21 [78]	*SIX3*
	14q22.1–22.3 [151]	*BMP4*
	ring21 [161]	?
